# Schwann cells in the inner ear: development, disease, and regeneration

**DOI:** 10.3389/fncel.2025.1662274

**Published:** 2025-09-10

**Authors:** Drew J. Montigny, Judith S. Kempfle

**Affiliations:** 1 Department of Otolaryngology, UMass Memorial Medical Center, Worcester, MA, United States; 2 University of Massachusetts Chan Medical School, Worcester, MA, United States; 3 The Eaton-Peabody Laboratories, The Massachusetts Eye and Ear Department of Otolaryngology - Head and Neck Surgery, Boston, MA, United States; 4 Harvard Medical School Department of Otolaryngology - Head and Neck Surgery, Boston, MA, United States

**Keywords:** Schwann cells, glia, inner ear, cochlea, schwannoma, NF2, regeneration, myelin

## Abstract

Schwann cells are classically known as the constituent supporting cells of the peripheral nervous system. Beyond the scope of merely myelinating axons of the more saliently known neurons, Schwann cells comprise the majority of peripheral nervous system tissue. Through the lens of the inner ear, additional properties of Schwann cells are becoming elucidated. Therein, the process of myelin formation in development is more aptly understood as a homeostatic oscillation of differentiation status. Perpetual interaction between neural and non-neural cells of the inner ear maintains an intricate balance of guidance, growth, and maturation during development. In disease, aberration to Schwann cell myelination contributes to sensorineural hearing loss in conditions such as Guillain-Barre Syndrome and Charcot-Marie-Tooth disease, and tumorigenic over proliferation of Schwann cells defines vestibular schwannomas seen in neurofibromatosis type 2. Schwann cells demonstrate plasticity during oscillations between differentiation and dedifferentiation, a property that is now being leveraged in efforts to regenerate lost neurons. Emerging strategies of reprogramming, small molecule modulation, and gene therapy suggest that Schwann cells could serve as progenitor cells for regenerated neurons. Understanding the duality of Schwann cells in pathology and repair could transform the approach to treating sensorineural hearing loss.

## 1 Introduction

Schwann cells are the fundamental glia of the peripheral nervous system, PNS. In the PNS, almost 80% of the cells surrounding the neurons are of glial origin (Rowitch and Kriegstein, 2010; Zuchero and Barres, 2015). Schwann cells are the supporting glial cells in the PNS and can be divided into myelinating and non-myelinating glial cells. Furthermore, satellite glia are a specialized subtype of peripheral glia, surrounding the neuronal cell bodies in the ganglia (Birren et al., 2025). Myelination by Schwann cells results in saltatory conduction, resulting in increased conduction velocity and temporal precision as compared to non-myelinated axons (Waxman, 1980; Salzer, 2015).

**Figure 1:**
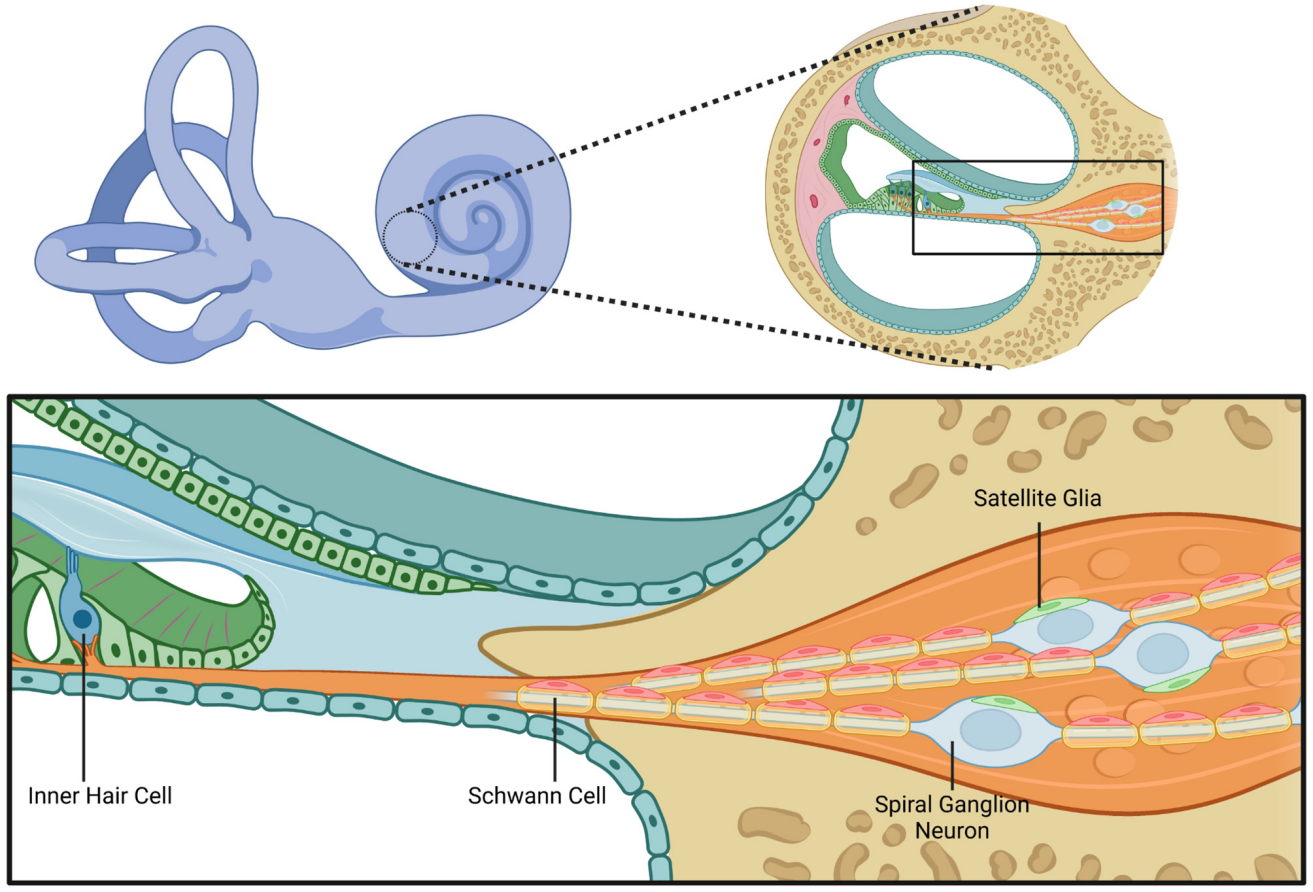
Cochlear structure and glial cell anatomy. This figure illustrates the anatomical structure of the cochlea with a focus on the cellular components involved in hair cell innervation by spiral ganglion neurons and the associated glial interactions. The left upper panel shows a schematic of the inner ear, highlighting the cochlea. The right upper panel shows a magnified cross-sectional view of the cochlear duct. The bottom panel provides a depiction of an inner hair cell within the organ of Corti and adjacent structures. The inner hair cell is innervated by the peripheral processes of spiral ganglion neurons. The peripheral processes are myelinated by Schwann cells. Within the spiral ganglion, neuronal cell bodies are surrounded by satellite glial cells. This figure highlights the spatial relationships between the inner hair cells that compose the sensory epithelium, spiral ganglion neurons, Schwann cells, and satellite glia. Created in BioRender. Montigny, D. (2025) https://BioRender.com/3b16cek.

Schwann cells are vital within the inner ear, an intricate sensory organ responsible for maintaining hearing and balance. Schwann cells within the cochlea provide structural and trophic support, supplementation of nutrients, and neurotransmitter recycling for auditory neurons that reside within the spiral ganglion, SGN, and vestibular neurons that reside with the vestibular ganglion, VN (also known as Scarpa's ganglia; Varon and Bunge, 1978). Schwann cells also play a role in the regeneration of damaged axons, first by clearing debris and by maintaining spatial arrangement guiding the regrowing axon to innervation (Jessen and Mirsky, 2019a). Auditory nerve fibers that span from inner hair cells in the organ of Corti all the way to the cochlear nucleus of the brainstem rely on Schwann cell myelination for temporally precise signal transmission to properly function (Figure 1).

## 2 Development

Schwann cells and neurons originate from a common progenitor in the ectoderm, indicating an intricate relationship. Understanding the developmental pathways of Schwann cells and neurons in the inner ear helps guide efforts to regenerate neurons of the SGN.

During the third week of embryonic development, the ectoderm thickens and begins to invaginate to form the otic placode. The otic placode further envelops into the otic pit, later developing into the otic vesicle. A portion of the epithelial layer of the otic vesicle, composed of neuroblasts, delaminates and migrates out of the otic vesicle and into the mesenchyme to later make up the SGN and VN whose peripheral processes innervate cochlear and vestibular hair cells, respectively, and whose central processes will make up the vestibular-cochlear nerve (Fritzsch et al., 2015; Alsina et al., 2009).

During the third week of human embryonic development, neurulation occurs in the ectoderm, which gives rise to the neural tube. The rostral portion of the neural tube is divided into six sections called rhombomeres (Bok et al., 2007). Ventral neural crest cells that migrate from the fourth rhombomere and differentiate into glial progenitor cells, also known as Schwann cell precursors, later differentiate into either myelinating Schwann cells that will myelinate the axons of SGN neurons, non-myelinating satellite glia that will reside within the SGN, or presynaptic Schwann cells (Alsina et al., 2009).

These neural crest-derived glial progenitors will move toward the recently migrated neuroblasts in the SGN and VN. Schwann cells migrate distally from SGN toward what will become the organ of Corti where they will myelinate the peripheral processes of SGN neurons (Alsina et al., 2009). Satellite glia will reside in their final location in the ganglia and will not migrate further (Alsina et al., 2009).

A distinct population of neural crest-derived glia form a passage to the cochlear duct to allow for the SGN neurons to innervate cochlear hair cells (Romand and Romand, 1990).

Immature Schwann cell precursors exist in proximity to both neuronal cell bodies and their processes. They both influence axonal pathfinding for peripheral axons and are influenced by their presence (Tylstedt et al., 1997).

Axonal activity and signaling influences Schwann cell differentiation (Moore and Linthicum, 2001). In the absence of Schwann cells, axons will still form and extend their processes, but they will extend past their destination and in various disordered directions (Mao et al., 2014). Schwann cell precursors act as intermediate targets for growing neurites (Sepp et al., 2001).

Neuronal processes and Schwann cell precursors create a scaffolding for which peripheral and central processes of these bipolar neurons that reside in the SGN can extend. Scaffolding is essential, and the glial precursors play a role in signaling for the axons to extend through this heterogeneous environment. Processes grow faster in the hind of the extending processes and the processes at the nerve front grow faster when in proximity to glia (Druckenbrod et al., 2020).

Sox10 is a known SRY-related HMG box family transcription factor expressed in differentiating and mature glia. In the mice lacking expression of Sox10, the peripheral processes of type 1 SGNs would extend past their peripheral target destination, the inner hair cells (Mao et al., 2014). This supports the findings of other studies that propose this mechanism where SCs guide and inhibit peripheral extension. Targeted conditional deletion of Sox10 caused peripheral extensions to extend past the inner hair cells and even to the lateral parts of the cochlea (Mao et al., 2014). However, central processes were not affected in the same way. Central processes were still successfully synapsing on their targets in the cochlear nucleus. This indicates central modulation that is not Sox10-dependent as it is in the periphery.

At gestational week 9, where the cochlea consists of one full turn, which will ultimately comprise the basilar turn, mature myelinating glia were located along central processes, within the ganglia around cell bodies and along peripheral processes (Locher et al., 2014). Sox10-positive glia were more densely located along the border of the SGN and not as prevalent throughout the center of the ganglia and the density of maturated myelinating glial cells was more pronounced centrally and decreased peripherally, indicative of a developmental wavefront. As this wavefront moves peripherally, Sox10-positive cells that remain in the SGN will most likely differentiate into satellite glia. Distinct from the glia that myelinate the central processes, the glia around the peripheral processes also expressed NGFR, nerve growth factor receptor. The presence of NGFR further implicates the existence of a developmental wavefront, implying that contact inhibition guides growing wavefront from the central aspect toward the periphery (Locher et al., 2014).

## 3 Myelination

Schwann and satellite cells, as they exist within the PNS, provide structural, metabolic, and trophic support to neurons through the mechanism of myelination (Varon and Bunge, 1978). Neurons depend on this support not only during development but also throughout the lifespan of the neuron.

Just as neurons depend on glia for survival, Schwann cell myelination is dependent on signaling pathways, some of which come from detecting normal neural signaling and temporally regular action potentials (Bouçanova and Chrast, 2020).

During promyelination, the stage immediately preceding full myelination, a temporally regulated epigenetic switch leads to the activation state of NF-kB, nuclear factor kappa-light-chain-enhancer beta subsequently leading to the expression of transcription factors including: Krox-20, also known as EGR2, Early Growth Response 2, Sox10, and Pou3F1, POU Class 3 Homeobox 1 (formerly Oct6; Chen et al., 2011). These transcription factors, induced by the activation state of NF-kB, lead to the production and promotion of myelinating proteins (Ghislain and Charnay, 2006). In response to upregulation of these transcription factors, Schwann cells begin to express myelin proteins such as P0, myelin protein zero, MAG, myelin-associated glycoprotein, and proteolipid protein 1 (Plp1; Fields, 2009). The promotion in the production of these proteins leads to molecular attraction both between the axon and myelin and between myelin and itself. Cellular adhesion molecules allow for the ensheathing of axons by myelin from Schwann cells in the PNS (Takeda et al., 2001; Garcia and Zuchero, 2019; Sukhanov et al., 2021). These extracellular signaling pathways play a role in tissue development. They are similar to the mechanisms discussed earlier that give rise to the anatomical structure of the ear during embryonic development.

Because myelination is inherently tied to normal and regular neural signaling, myelinating Schwann cells downregulate myelin-associated gene expression in the absence of neural activity and dedifferentiate into a promyelinating or non-myelinating phenotype (Momenzadeh and Jami, 2021). In the case of the inner ear of ototoxic drug-deafened mice, Schwann cells myelinating peripheral and central processes of SGNs downregulated P0 (Hurley et al., 2007).

**Figure 2:**
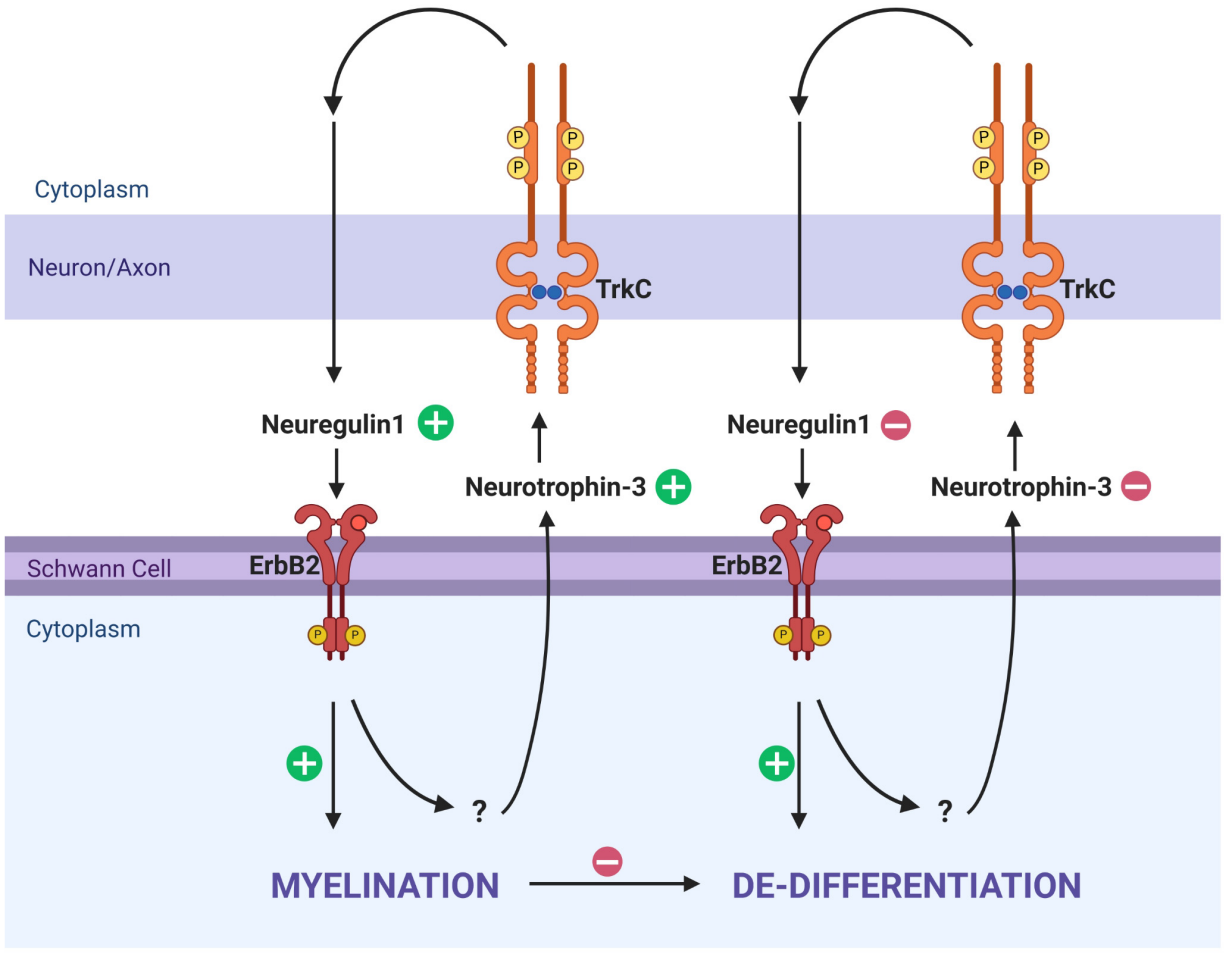
Myelination and dedifferentiation via ErbB2 and TrkC signaling. This schematic illustrates molecular signaling pathways that influence Schwann cell myelination and dedifferentiation in response to axonal feedback. Neuregulin1 and Neurotrophin-3 are key ligands interacting with ErbB2 on Schwann cells and TrkC on axons, respectively. On the left side, Neurotrophin-3 from Schwann cells binds TrkC on axons, leading to Neuregulin1 signaling from axons which will then bind ErbB2, reinforcing myelination in a homeostatic manner. When this process is disrupted, the Schwann cell will trend toward dedifferentiation, and no longer produce Neurotrophin-3. TrkC is likewise not bound, and the axon does not produce Neuregulin1, subsequently not binding ErbB2, reinforcing the de-differentiated state. Created in BioRender. Montigny, D. (2025) https://BioRender.com/gdz3ihk.

The myelination status of Schwann cells in the PNS is most commonly understood in terms of progression from undifferentiated to differentiated, whereas it could best be understood as an oscillation between differentiated (myelinating), dedifferentiation (non-myelinating), and redifferentiation (myelinating) depending both on developmental processes and pathological processes (Salzer, 2015). Neuregulins are a family of epidermal growth factors that provide trophic support in neuron–glia interactions in the CNS and PNS (Falls, 2003). In the periphery, sensory neurons in the ganglia express Neuregulin1 (Nrg1; Rio et al., 1997). Nrg1, expressed by neurons, binds ErbB2, v-erb-b2 avian erythroblastic leukemia viral oncogene homolog 2, on Schwann cells (Rio et al., 1997). A transgenic mouse model expressing double negative ErbB2 receptors led to significant neuronal loss without loss of sensory hair cells, suggestive of a feedback loop between SGN neurons and Schwann cells (Stankovic et al., 2004). In this model, Schwann cells significantly downregulated neurotrophic factor 3 (NT3), while significantly upregulating glial-derived neurotrophic factor (GDNF; Sugawara et al., 2007; Stankovic et al., 2004). A recent *in vitro* study that deprived Schwann cells of Nrg1 significantly upregulated ErbB2 receptors (Chiasson-MacKenzie et al., 2023). NT3, expressed by Schwann cells under homeostatic conditions, binds to TrkC receptors on SGNs and plays a key role in neuronal survival, neurite outgrowth, and maintenance. The loss of NT3 following ErbB2 inhibition suggests that Nrg1/ErbB2 signaling is required to maintain NT3 expression. In contrast, upregulation of GDNF may represent a response by the Schwann cells to preserve remaining SGNs. These findings demonstrate that the Nrg1/ErbB2 axis may not only be implicated for structural myelination but also shift the environment from homeostatic to reactive (Gómez-Casati et al., 2010; Kohrman et al., 2021). This interplay between sensory neurons and Schwann cells is one feedback method they use to remain in homeostasis (Newbern and Birchmeier, 2010; Figure 2).

Axonally expressed N-cadherin and beta-catenin play a role in Schwann cell proliferation and differentiation. *N-cadherin* is a cell adhesion molecule that is present on the surface of the axon. When N-cadherin was bound by an inhibitor, Schwann cells decreased association with the axon and reduced proliferation. In addition, reduction of beta-catenin, the binding partner of N-cadherin, significantly decreased Schwann cell proliferation despite the addition of mitogenic Heregulin-beta1 (a synthetic homolog to Nrg1), suggestive of a relationship between Nrg1/ErbB2 and beta-catenin dependent WNT proliferation (Gess et al., 2008).

The extracellular matrix also plays a role in the myelination status of Schwann cells in the PNS. Early in the differentiation of Schwann cells, laminin binds beta1 integrins on the cell surface, leading to the assembly of basement membranes necessitated prior to the development of myelination. Laminin/integrin interactions lead to scaffolding formation and cell adhesion secondary to the basement membrane formation (McKee et al., 2012).

Secondary messenger systems also influence the myelination of Schwann cells via G-protein coupled receptor 126 (Gpr126). To date, the ligand that binds to Gpr126 is unknown, although it is likely signaling from the axon (Glenn and Talbot, 2013a; Lemke and Chao, 1988). During the promyelination stage, Schwann cells are activated via the Gpr126 cAMP pathway that leads to expression of transcription factor Pou3F1 and Pou3F2, POU Class 3 Homeobox 2 (Oct6 and Brn-2; Jaegle et al., 2003). Gpr126 is essential for the initial expression of these transcription factors, leading to the expression of Egr2, yet Gpr126 is not needed for sustained myelination (Glenn and Talbot, 2013a).

Both Oct6 and Nrg1 are needed for remyelination following injury (Fricker and Bennett, 2011; Fricker et al., 2011). In the post-injury environment, mammalian target of rapamycin complex 1 (mTORC1) activation is needed to drive dedifferentiation in Schwann cells through increasing translation of cellular Jun proto-oncogene (c-Jun) mRNA. Prolonged mTORC1 activation led to prolonged time to remyelination (Norrmén et al., 2018). Similarly, the Schwann cells remained dedifferentiated so long as mTORC1 was activated, allowing redifferentiation and remyelination upon mTORC1 downregulation (Norrmén et al., 2018).

## 4 Non-myelinating peripheral glia of the inner ear

The major constituent non-myelinating peripheral glia of the inner ear are satellite glia. These non-myelinating glia play a role in the inner ear similar to that of glia in the CNS in terms of metabolic and trophic support. Satellite glia around SGN neurons have membrane proteins that allow a slight inward leak of ions around the time of hearing onset, which may indicate an alternative role for these glia (Smith et al., 2021). Potentially, these glia foster a cellular environment that will maintain homeostasis while potentiating neural pathways during the onset of hearing. Satellite glia that ensheathe the soma of peripheral neurons uphold intracellular neural pathways that lead to neurite regeneration (Avraham et al., 2020). The satellite glia within the inner ear, specifically a particular population of Sox2, sex-determining region Y (SRY)-box 2, non-myelinating satellite glia, can serve as progenitors for neural regeneration (Chen et al., 2021).

Myelination of neuronal cell bodies is uncommon across the whole PNS in human. In neonatal humans, myelination of the soma of SGN neurons has not been observed. Some myelination within the SGN has been reported in humans, with an age-associated increase in ganglionic myelination, albeit, at most only a few lamellae are observed to surround the SGNs in human. However, at its maximum in the elderly, a mere 2% of SGN somas are associated with myelination by satellite glia (Arnold, 1987; Nadol et al., 1990). To date, there is no accepted consensus on why the human cochlea lacks neuronal soma myelination by satellite glia. When comparing myelination within the SGN between humans and non-human mammals, expression levels of myelin-basic protein (MBP) demonstrated a lack of organized myelination in humans (Liu et al., 2012). In contrast, regularly patterned myelination by satellite glia has been observed in mice, rats, and guinea pigs (Nadol et al., 1990). Schwann cells that myelinate the axon provide axonal trophic support while satellite glia that myelinate the soma provide somatic trophic support. The SGN's fortitude depends on the presence of presynaptic peripheral innervation of the hair cells and postsynaptic transmission through the cochlear nerve (Alam et al., 2007; Spoendlin, 1975). Normal signaling necessitates receiving and transmitting signals through both peripheral processes myelinated by Schwann cells and central processes myelinated by oligodendrocytes. In non-human mammals, satellite glia within the ganglia provide the necessary trophic support and maintain electrical signaling necessary for the survival of the neurons. There are cross-species associative differences between SGNs; in non-myelinating human SGN, neurons closely associate into a patterned formation, whereas in non-human mammals, the structure appears to be provided by myelinating satellite glia within the ganglia (Liu et al., 2012). There must be some advantage that the lack of myelination provides to these neurons. One possible explanation suggests that SGNs have evolved to associate closely with each other intentionally (Jyothi et al., 2010). The lack of myelination and close association may confer an advantage during afferent apoptosis, or hair cell death (Jyothi et al., 2010).

This increases robustness of SGN survival secondary to hair cell loss in the human SGN as compared to other non-human mammals (McFadden et al., 2004; Suzuka and Schuknecht, 1988; Zilberstein et al., 2012). The somas of human SGNs lose the trophic and metabolic support from the myelination of glia within the ganglia. However, they gain potential for electrical coupling and avoid apoptotic pathways due to insufficient signaling. If it is true that there are no myelinating satellite glia in the human SGN, then there may be alternative pathways and mechanisms unique to humans that are not seen in other mammalian models (McFadden et al., 2004; Suzuka and Schuknecht, 1988; Zilberstein et al., 2012).

Within the spiral ganglia, there are two types of SGNs. Type 1 SGNs have large myelinated fibers that relay sensory information from inner hair cells and compose approximately 95% of SGNs (Spoendlin, 1971). Type 2 SGNs have small unmyelinated fibers that relay output to outer hair cells to modulate and attenuate cochlear amplification and compose about 5% of SGNs (Spoendlin, 1971). The difference in myelination is likely necessitated for temporal precision of incoming auditory information by Type 1 SGNs. Interestingly, following transection of the cochlear nerve, Type 1 SGNs exhibit retrograde degeneration while Type 2 SGNs do not degenerate. This evidences that Type 1 SGNs are dependent on target mediated trophic support and continual synaptic activity (Spoendlin, 1971).

## 5 Sensorineural hearing loss and myelination related to aging

Sensorineural hearing loss, SNHL, is hearing loss related to damage or degeneration of inner ear starting anywhere from inner hair cells onto pathways connecting the inner ear to the brain, as juxtaposed to conductive hearing loss related to the middle and outer ear. Presbycusis, age-related hearing loss, was traditionally defined by the loss of SGN neurons secondary to hair cell loss (Elliott et al., 2022). Reversible threshold shifts with no hair cell loss still cause a substantial permanent loss of synapses.

Schwann cell pathology, as seen in aging, is due to a reduction in myelin maintenance and thinning of the myelin sheath that imparts a loss of regenerative and communicative capacity to their neural counterparts, as well as an increased vulnerability to environmental stressors (Glenn and Talbot, 2013b; Stassart et al., 2013).

Schwann cells myelinating auditory nerve fibers also demonstrate age-related deterioration. Schwann cells and satellite glia provide trophic support for neurons. Age-related aberration and loss of myelination, represented by depleted expression levels of MBP, myelin basic protein, in aging human and mouse cochlea has been observed (Lang et al., 2011). Even a transient bout of demyelination can elicit a lasting hidden hearing loss (HHL) phenotype (Wan and Corfas). HHL refers to hearing loss and abnormalities that are difficult to diagnose with typical audiogram style hearing tests and usually requires testing of extended high frequencies, distortion product otoacoustic emissions and auditory brainstem responses (DPOAEs and ABRs), respectively. HHL presents with normal pure tone audiogram thresholds but abnormal ABR data (Wan and Corfas, 2017). ABRs also known as auditory brainstem response are an electrical waveform representative of neural conduction encoding sound as it passes from the cochlea to the brain. Peak one of the ABR represents the specific activity of the SGNs (Jewett and Williston, 1971). Peak one amplitude represents the summative potential of the SGNs action potentials, where selective ablation of SGNs with ouabain decreases peak one amplitude (Yuan et al., Lang et al., 2008). Peak one latency is inversely proportional to conduction velocity and by logic the health of myelinating Schwann cells (Jewett and Williston, 1971; Salzer, 2015). There is a decrease in peak one amplitude in aging humans and mice as compared to their younger counterparts (Lang et al., 2011; Xing et al., 2012). It remains unclear whether this decrease in peak one amplitude occurs prior to the neural loss and is mutually exclusive of hair cell loss. Age-aberrated myelination offers a potentially explanatory pathway in which myelin sheath loss occurs prior to neural degeneration and is an area of future exploration. Based on ultrastructural study finding that the vast majority of human SGN neurons lack myelination at the soma, the fact that age-associated loss of neurons in all mammals is suggestive that neural degeneration may occur primary to secondary myelination aberration (Nadol et al., 1990; Lang et al., 2011). Age-related myelin deficits offer an explanation for some cases of HHL wherein loss of myelination and conductance velocity confers perturbed temporal processing and appraisal (Kohrman et al., 2021).

## 6 Schwann cell disorders

Schwann cells play a significant role in the pathology of the inner ear. On one end of the spectrum, over proliferation of Schwann cells in the vestibulo-cochlear nerve forming vestibular schwannomas leads to hearing loss in diseases such as neurofibromatosis type 2-schwannomatosis (NF2-SWN), while demyelinating disorders, such as in Charcot-Marie-Tooth Disease Type 1A, CMT1A, or Guillain-Barre Syndrome (GBS), are associated with hidden hearing loss (Wan and Corfas, 2017; Cassinotti et al., 2024). Schwann cells uphold the delicate balance within the inner ear; their range of functionalities discussed thus far carries the converse during dysregulation.

### 6.1 Myelinopathies

When discussing the importance and intricate balance Schwann cells maintain in the inner ear, it would be remiss not to mention demyelinating disorders and their role in inner ear pathology. GBS is an acute autoimmune demyelinating disorder. Onset typically occurs after respiratory or digestive illness; although the exact pathophysiology remains unknown, but for functional purposes, T lymphocytes lose autoimmune tolerance and attack peripheral nerve components, including Schwann cells and their myelin sheaths (Dalakas, 2013).

Transient loss of myelin in the periphery, specifically the Schwann cells that myelinate the SGN peripheral processes, is associated with HHL characteristic symptoms such as the drastic increase in peak one latency and decrease in peak one amplitude despite otherwise normal ABR/DPOAE (Wan and Corfas, 2017). These changes were permanent despite the Schwann cells remyelinating the peripheral processes.

Patients with Charcot-Marie-Tooth Disease Type 1A have similar pure tone audiograms as compared to age matched controls; however, speech in-noise testing revealed a significant difference wherein the CMT1A patients had much more difficulty understanding speech in noise. The difference was accounted to a reduction in temporal resolution associated with an aberration in the Schwann cell myelination (Cassinotti et al., 2024; Choi et al., 2018).

A mouse model of CMT1A confirmed little to no loss of auditory nerve fibers yet disruptions in myelination at the heminodes were associated with the HHL phenotype observed in patients with CMT1A (Cassinotti et al., 2024).

It was discerned that this mechanism was independent of synaptopathy as the effects from hair cell ablation were additive to the changes from acute demyelination. This, in addition to findings from Charcot-Marie-Tooth Disease, system lupus erythematosus and other episodes of acute demyelination both support and necessitate the need for homeostatic Schwann cell myelination in the inner ear. In the cases of Schwann cell demyelination due to GBS and systemic lupus erythematosus, the immune system, specifically the quiescent macrophage phenotype, is the purported culprit (Choi et al., 2018; Berger et al., 2006; Griffin and Sheikh, 1999; Di Stadio and Ralli, 2017; Budak et al., 2021, 2022). There is some evidence that this is analogous to multiple sclerosis by way of oligodendrocytes in the CNS regarding abnormal latencies on auditory brainstem responses (Furst and Levine, 2015).

### 6.2 NF2 and schwannomatosis

Schwannomas that frequently present in patients with NF2-SWN are tumors of the nerve sheath known as vestibular schwannomas, or formerly acoustic neuromas. Pertinent to the inner ear, the presence of vestibular schwannomas can lead to hearing loss, loss of balance, tinnitus, and vertigo. Patients with vestibular schwannomas present with hearing loss as the first symptom in approximately 90% of cases, with high frequency SNHL being the most prevalent (Harner et al., 2000). As the vestibular schwannoma progresses, it will put pressure on surrounding areas. Outgrowth that compresses the brainstem is fatal. Both natural growth and surgical treatment can also damage the facial nerve which lies in the surrounding area. In many cases, this leads to facial paralysis, numbness, or paresthesia (MacCollin et al., 2005; Vakharia et al., 2012). NF2-SWN is due to an autosomal-dominant germline mutation in the NF2 gene. The NF2 gene, a tumor suppressor gene, normally codes for a protein known as Merlin, also known as moesin-ezrin-radixin-like-protein, schwannomin or neurofibromin 2. As a normally functional tumor suppressor protein, Merlin inhibits several key proliferative and expansive pathways in Schwann cells. Merlin was traditionally considered a cytoplasmic scaffolding protein that provided mechanistic information from the extracellular environment to intracellular cytoplasmic pathways. There is varying utilization and intracellular signaling with regard to these growth factors (McClatchey and Giovannini, 2005). In addition, the loss of Merlin in NF2 leads to a lack of growth inhibition and tumorigenesis. Similarly, Merlin does, in fact, function to inhibit mitogenic pathways. In the functional pathway, Merlin migrates to the nucleus to elicit tumor-suppressive gene expression (Li et al., 2012; Beltrami et al., 2013; Ren et al., 2021).

Schwannomatosis as a disease is characterized by uninhibited proliferation of Schwann cells, inevitably giving rise to a tumor known as schwannoma. Schwannomatosis not related to NF2 can result from mutations in tumor suppressor genes on chromosome 22 including but not limited to the SWI/SNF-related BAF chromatin remodeling complex subunit B1 (SMARCB1) and leucine zipper like post-translational regulator 1 (LZTR1 genes; Tamura, 2021). As a disease, it is classified by at least one schwannoma confirmed pathologically and a common variant of SMARCB1, LZTR1, or loss of heterozygosity at chromosome 22q in two anatomically separate schwannomas and without the presence of bilateral vestibular schwannomas. However, the current criteria do not exclude unilateral vestibular schwannomas or meningiomas (Plotkin et al., 2022). The presence of bilateral vestibular schwannomas is rather indicative of NF2-SWN. Patients with unilateral vestibular schwannomas have ipsilateral SNHL in approximately 20% of cases and are at an increased risk for developing moderate hearing loss in the contralateral ear, suggestive of underlying genetic predisposition and may serve as evidence of tumor secretions although these mechanisms remain ill defined (Sauvaget et al., 2005).

Histologically, peripheral nerve sheath tumors such as schwannomas are described to maintain two distinct extracellular architectures, which can be seen as recognizable patterning: Antoni A and Antoni B. Antoni A is characterized by hypercellularity. Antoni A regions of schwannomas are organized in a stacked wave-like fashion known as a palisade. Antoni A patterning is uncommon in vestibular schwannomas. Antoni B, in contrast, is hypocellular and is most common in VS. Extracellular architecture includes regions of acellularity called Verocay bodies and microcysts. Unlike Antoni A, the extracellular environment of Antoni B supports the activation of lymphocytes and microglia. Antoni B areas foster an environment susceptible to immune cell infiltration (Labit-Bouvier et al., 2000). Immune cells, specifically tumor-associated macrophages, foster a pro-inflammatory cascade that drives tumorigenesis via angiogenesis as well as chemokine/cytokine signaling (Hannan et al., 2020). A study comparing the proliferative potential of schwannomas identified that MIB-1-defined proliferation mostly occurred at the transition zone of Antoni A and B areas alongside the presence of macrophages. However, mitosis was only noted as positive in Antoni A areas (Abe et al., 2000). In an earlier study, Antoni B areas were ridden with signs of degeneration, including fragmented and detached basal lamina, disrupted tissue, and acellularity (Sian and Ryan, 1981). Schwann cell-to-neuron feedback loops of contact and contact inhibition are integral in maintaining healthy myelination and homeostasis (Bouçanova and Chrast, 2020; Cristobal and Lee, 2022).

Schwann cells secrete TNF-alpha, a pro-inflammatory cytokine (Wagner and Myers, 1996). The Schwann cell release of TNF-alpha related to a NF-kB-derived pathway (Qin et al., 2012). NF-kB signaling stimulates the release of pro-inflammatory cytokines and chemokines, and NF-kB pathways promote cell proliferation while inhibiting apoptosis (Liu et al., 2017). NF-kB promotes the growth of schwannomas (Elmaci et al., 2018). Merlin inhibits the NF-kB signaling pathway (Kim et al., 2002). NF-kB-mediated tumorigenesis is evidenced through results focused on attenuating cyclooxygenase 1 pathways. Tumor growth was reduced in patients taking acetylsalicylic acid (Kandathil et al., 2014; Dilwali et al., 2015). The NF-kB pathway may be the driving force of schwannoma proliferation.

Vestibular schwannomas almost always arise on the vestibular portion of the vestibulo-cochlear nerve in the internal auditory canal or cerebellar pontine angle (Sataloff et al., 1988; Gosselin et al., 2016; Zhang et al., 2022; Choayb et al., 2023; Khrais et al., 2008). Cases of intralabyrinthine schwannomas have been reported, but the origination of these Schwannomas remains for debate (Zhang et al., 2022). Hearing loss associated with vestibular schwannomas was historically understood as mechanical compression of the cochlear nerve leading to aberration in neural transduction which was realized as hearing loss. However, hearing loss develops regardless of tumor size or tumor growth (Brown et al., 2022). Some degree of hearing loss seen with vestibular Schwannomas may be due to mass effect as seen with meningiomas and other masses in close proximity to auditory pathway (Ruiz-Garcia et al., 2024).

**Figure 3:**
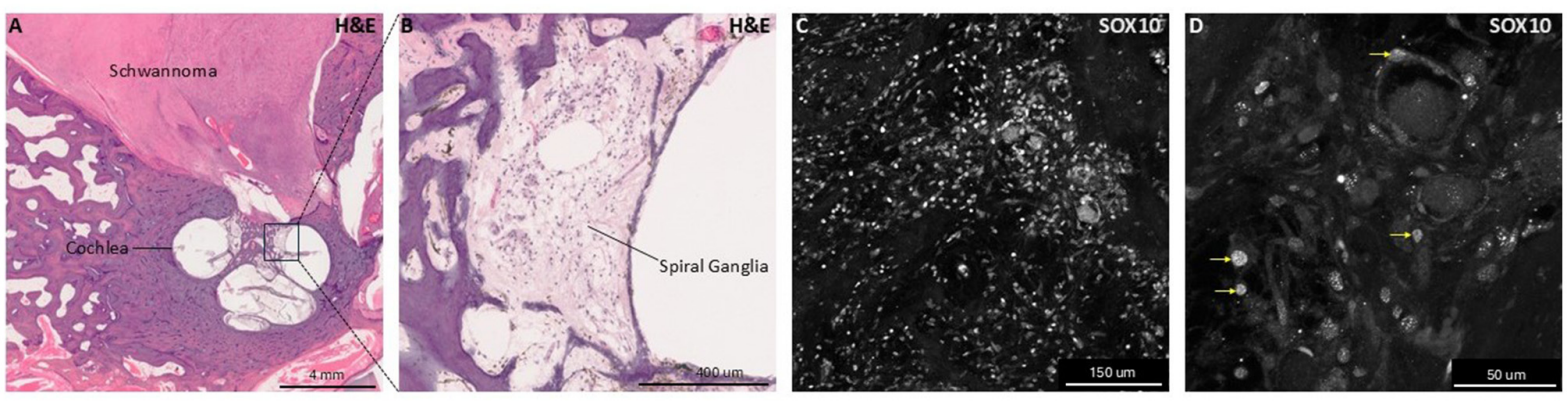
Histologic and immunohistochemical human temporal bone samples with Schwannoma involvement. This figure presents hematoxylin and eosin (H&E) stained and SOX10 immunolabeled images for human temporal bone sections highlighting glial pathology and anatomy. Panel **(A)** shows a low-magnification H&E-stained section of a human temporal one containing a vestibular schwannoma from a patient with neurofibromatosis type 2-associated schwannomatosis (NF2-SWN) occupying the internal auditory canal, illustrating schwannoma localization to inner ear structures. Panel **(B)** shows higher magnification view focusing on the spiral ganglion, where spiral ganglion neurons and glia reside. Panels **(C, D)** show immunofluorescent labeling of SOX10, a nuclear glial cell marker, within the spiral ganglia. Panel **(D)** includes yellow arrows indicating examples of SOX10+ nuclei, emphasizing the presence and distribution of glial cells within the ganglia. These images demonstrate both histological architecture of glial cell populations and pathological human cochlear tissue.

Patients with NF2-SWN present with peak one latency and decrease of peak one amplitude in ABR (Thomsen et al., 1978; Musiek et al., 1980). The audiological abnormalities observed in the ABRs of NF2-SWN patients parallel changes in ABRs seen in cases of auditory nerve demyelination (Wan and Corfas, 2017). Given that vestibular schwannomas arise from Schwann cell origins, aberration of Schwann cell myelination could serve as the etiological basis for hearing loss in NF2 (Figure 3). These findings suggest that hearing loss with vestibular Schwannoma may not only involve neural compression but a reprogramming of Schwann cells that may coincide with tumor onset and progression (Mohamed et al., 2023).

## 7 Inner ear Schwann cells and regeneration

SNHL in humans and mammals has historically been viewed as irreversible due to the lack of spontaneous repair or renewal of damaged cellular structures, including hair cells, synapses, and neurons. However, two decades ago, the discovery of progenitor-like cells within the cochlea opened up new possibilities for hearing restoration. While these dormant cells initially claimed limited regenerative potential *in vivo*, they demonstrated promise for regeneration when stimulated *in vitro* (Li et al., 2003).

The inner ear regeneration field has since focused on three main goals: identifying and characterizing inner ear progenitors, identifying manipulators to initiate cell fate switches, and increasing the yield of newly generated hair cells.

SNHL is linked to the loss of hair cells and/or neurons, which has been linked to age- and noise-related hearing loss, as well as ototoxic and genetic factors. Unlike mammals, non-mammalian species such as amphibians and birds can spontaneously regenerate hair cells through the continuous division and transdifferentiation of supporting cells. Researchers have aimed to understand the molecular mechanisms underlying hair cell regeneration in these species to apply similar principles to the mammalian inner ear. Supporting cells are developmentally related to hair cells and provide trophic support during development and throughout life. After damage to hair cells, supporting cells can be stimulated and coaxed to differentiate into hair cells.

Inner ear Schwann and satellite cells have many similarities to supporting cells; these glia and inner ear neurons share a distant common progenitor, and inner ear glial cells provide trophic support for normal neuronal function during development, aging, and disease (Delaunay et al., 2008; Doetsch, 2003; Rowitch and Kriegstein, 2010). Glial cells of the CNS can be coaxed into becoming neurons upon forced overexpression of pro-neural genes, i.e., Achaete-scute homolog 1 (Ascl1) and Neurogenin 1 (Ngn1; Jessen and Mirsky, 2019a; Davis and Temple, 1994). Astrocytes are implicated to modulate neurogenesis, synaptic reorganization, and neural plasticity overall in the CNS.

Overexpression of transcription factors Paired Box Gene 6 (Pax6) and Neurogenin 1 and 2 induce astrocytic conversion to glutamatergic neurons, while Ascl1 and Dix1/2 were able to convert astrocytes to GABAergic neurons (Guillemot, 2005; Schuurmans et al., 2004; Casarosa et al., 1999; Parras et al., 2002). Albeit there was CNS region-specific ability to convert to particular neuronal subtypes and lack of evidence of a functional readout, epigenetic modulation of the CNS was able to reprogram astrocytes to neural phenotypes.

In the post-injury environment in the CNS, there is a period known as reactive gliosis, which includes a proliferation of reactive astrocytes (Robel et al., 2011). During this proliferation, reactive astrocytes upregulate Sox2, a neural stem cell marker, and eventually differentiate into fully functional neurons (Niu et al., 2015). After this proliferation, the glia form what is known as a glial scar in the nervous tissue, which functions to prevent the spread of inflammation through microglial invasion and creates a border to limit the bounds of fibrotic tissue (Yang et al., 2020). Glial scarring, however, inhibits the outgrowth of reforming neural processes both through the formation of a fibrous scar and cytokine release (Kimura-Kuroda et al., 2010).

After neuronal damage or loss in the PNS, Schwann cells and satellite cells remain within the cochlea and usually survive long term, but rather than regenerating into neurons, only a glial scar is initiated (Yuan et al., 2014). Both in spinal cord injury and facial nerve injury, Schwann cells contribute to the regenerative process by migrating toward the site of damage, proliferating, and guiding damaged axon's regrowth by providing cellular scaffolding (Jessen and Mirsky, 2019b; Wang et al., 2022), although maladaptive Schwann cell proliferation and gliosis during the post-injury stage may ultimately hinder functional recovery due to glial scarring (Jessen and Mirsky, 2019b; Wang et al., 2022).

Following loss of sensory epithelium in deafened rat cochlea, Schwann cells dedifferentiated to non-myelinating phenotypes (Hurley et al., 2007). Recent work indicates that, if properly stimulated, these glial cells may also exhibit regenerative potential (Lang et al., 2011). Immediately after primary degeneration of the inner ear, Schwann cells upregulate the transcription factor Sox2 (Lang et al., 2011). Similarly, the Sox2-positive phenotype of these Schwann cells mimics the Sox2-positive neural progenitor phenotype of astrocytes in the CNS. Furthermore, the Sox2-positive population of Schwann cells that resides in the SGN, *in vitro*, can be reprogrammed to become phenotypical neurons (Chen et al., 2021).

During the development of the inner ear, expression of pro-neural transcription factor Ngn1 and basic helix-loop-helix, bHLH, transcription factor neuronal differentiation 1, NeuroD1 drive neural differentiation (Jeon et al., 2011; Evsen et al., 2013). In the CNS, expression of NeuroD1 and Ngn1 is sufficient to drive neural conversion from glia, while expression of NeuoD1 and Ascl1 drove neural conversion from astroglia. In an injury model, overexpression of pro-neural genes in glia was necessary for glia-to-neuron conversion (Heinrich et al., 2014; Su et al., 2014).

Subsequently in the same fashion, explanted Schwann cells from the SGN were grown in cell culture and, when treated with specific substrates, would develop into what is now referred to as neurospheres (Diensthuber et al., 2014). Ensuing ideas based on hair cell regeneration from supporting cells looked to see if the same could be accomplished for turning Schwann cells toward a neurogenic fate. *In vitro*, explanted Plp1-positive glia spontaneously differentiated into neurons and began the outgrowth of neurites, even forming synapses (Martinez-Monedero et al., 2007; Guo et al., 2009; Le Bras et al., 2005; McLean et al., 2017; Shi and Edge, 2013; Denton et al., 2016).

Within the SGN of the inner ear, there is a population of early neural crest progenitor cells that express Plp1 (Li et al., 2003; McLean et al., 2016; Breuskin et al., 2010; Harlow et al., 2014;; Kempfle et al., 2020).

However, within the bounds of the post-injury environment, Sox2-positive, or more broadly Plp1-positive, Schwann cells within the SGN can be reprogrammed into neurons through upregulation of key transcriptional factors and a variety of small molecules. Sox2 and c-Jun coregulate myelination negatively in human schwannomas, pushing Schwann cells toward a dedifferentiated state (Shivane et al., 2013). Unlike Schwann cells in the somatic PNS, where injury induced dedifferentiation supports axonal regeneration over long distances, Schwann cells in the inner ear rarely support axonal regrowth *in vivo* despite transcriptional changes. While c-Jun upregulation is common to both environments, the outcome of regenerated axons is far more limited in the cochlea. There may be differences in extracellular matrix, absence of permissive structural scaffolds, or suppression of inflammatory cascades needed for axonal regrowth or remyelination.

The pertinent Sox2-positive cells upregulate key neuronal factors such as Ascl1 or high mobility group A2 (Hmg2a). As they convert to a more neural phenotype, Sox2 expression is downregulated while Ascl1 remains upregulated.

Similarly, an RNA-binding protein Lin28 promotes the proliferation of neural precursors during early murine development. At later time points, Lin28 interacts with Let7 microRNA to promote differentiation of these proliferating progenitor cells. In addition, Lin28 upregulated Hmg2a, which was upregulated in Sox2-positive cells. Similarly, Lin28 maintained Plp1 progenitor cells in the inner ear and promoted neuronal differentiation (Cimadamore et al., 2013; Newman et al., 2008; Viswanathan and Daley, 2010; Heo et al., 2008). Ngn1 drives the expression of NeuroD1 when activated by RNA binding protein Lin28 (Kempfle et al., 2020). In conjunction, Let7 induction via the Lin28/Let7 axis decreases the Hmg2a neural stemness and allows the cell to express and upregulate neural-derived bHLH transcription factors Ngn1 and Ascl1 (Cimadamore et al., 2013; Kumar et al., 2022), pushing the cells to neural fate through facilitating cell cycle exit via upregulation of post-mitotic pro-neural genes. To this day, it remains unclear, which subpopulation of glial cells in the inner ear truly harbors the most regenerative potential for neuronal regeneration. The search for “dormant” glial progenitor cells in the inner ear continues, with the hope of further enhancing the understanding and potential for inner ear neuron regeneration (Kempfle et al., 2020).

## 8 Potential gene and cell therapy approaches for SNHL

Gene therapy is gaining traction for regeneration of neurons. Current strategies involve viral vectors, i.e., adeno-associated viruses (AAVs), to deliver reprogramming factors and modulate gene expression mainly targeted at the transcription factors Ascl1, Sox2, and NeuroD1.

**Figure 4:**
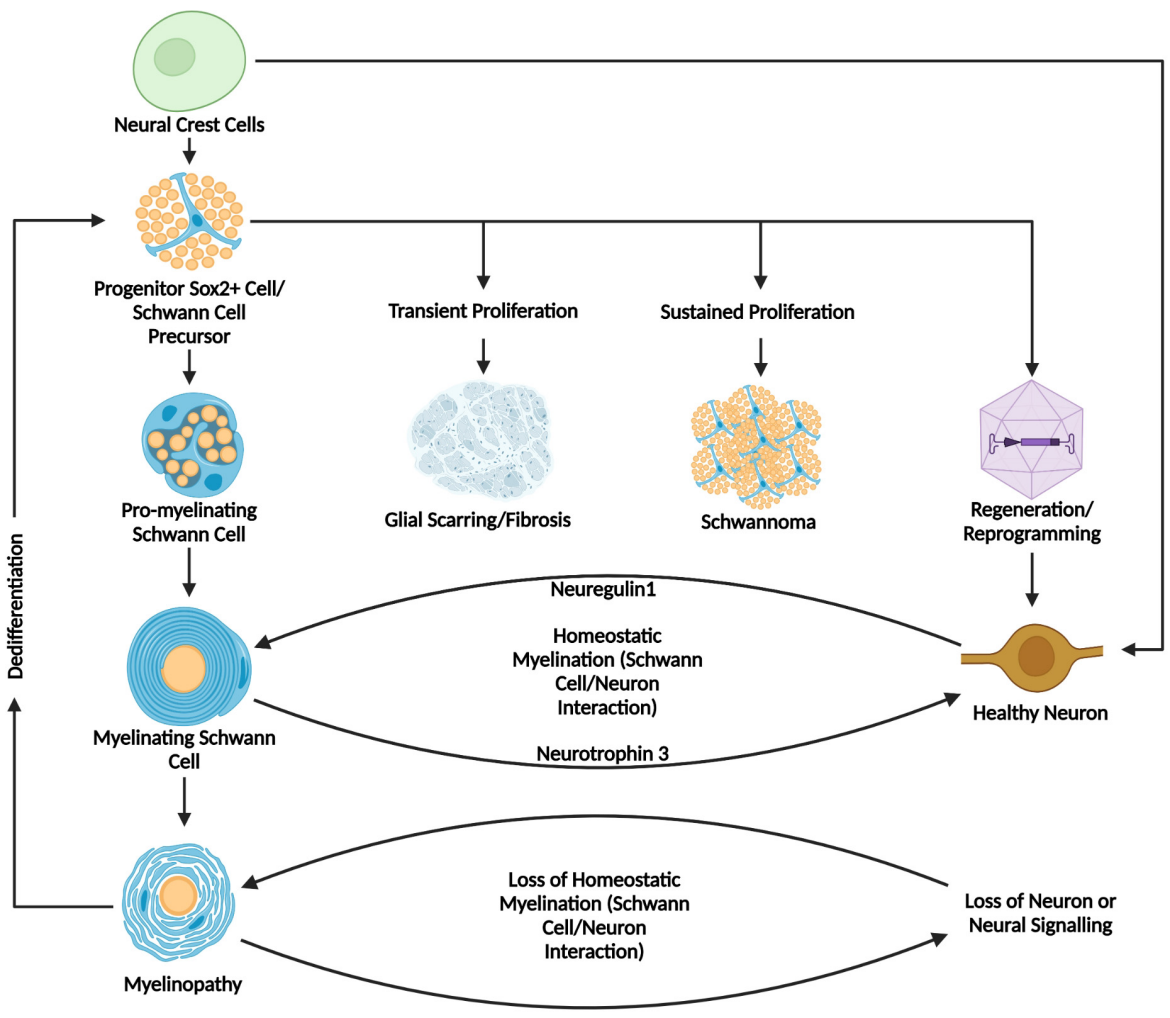
Summary diagram of Schwann cell development, myelination, and pathology in homeostasis and disease. This figure summarizes the developmental origins, pathways of differentiation, and potential outcomes of Schwann cells, highlighting their dynamic roles in homeostasis and pathology. Starting from neural crest cells and later progressing to Schwann cell precursors before developing further to pro-myelinating and mature myelinating Schwann cells. Healthy myelinating Schwann cells interact with neurons through a Neuregulin1/Neurotrophin-3 mediated axis, while myelinopathy or loss of healthy neural tissue can disrupt this feedback loop. Downstream outcomes in pathological states can lead to myelinopathy and subsequent de-differentiation leading to a less differentiated state. It is in this state that Schwann cells can proliferate transiently, leading to formation of a glial scar, or proliferate continuously leading to a schwannoma, or alternatively, be prompted toward a neural fate through cellular reprogramming or transdifferentiation. Created in BioRender. Montigny, D. (2025) https://BioRender.com/s9fe79f.

Various small molecule drugs have been used in combination with viral vectors to increase neural yield in conversion in both the PNS and CNS that may serve as a more clinically approachable solution to reprogramming neurons in the inner ear of humans (Pfisterer et al., 2016; Wapinski et al., 2013; Treutlein et al., 2016; Mall and Wernig, 2017; Velasco et al., 2017; Ma et al., 2019). Histone modifiers: molecules such as Trichostatin A and Valproic acid alter histone acetylation increasing chromatin accessibility lending toward neural reprograming (Smith et al., 2016). Compounds such as forskolin, CHIR99021(a GSK-3 inhibitor), and ISX-9 leverage signaling pathways toward neuronal differentiation through transcriptional level changes, i.e., the ability to modulate transcription factor binding (Wapinski et al., 2013; Pfisterer et al., 2016; Yin et al., 2019; Figure 4).

The potential for Schwann cells in the inner ear to serve as a source of regenerative neural progenitors represents a novel conceptual shift in the field. Traditionally known as purely supportive, emerging evidence suggests that Schwann cells may be harnessed for neural reprogramming and transdifferentiation. Schwann cells in the inner ear exhibit the capacity for plasticity and position themselves to not only contribute to disease but to be the target for the future of therapeutic strategy in SNHL (Meas et al., 2018).

## 9 Conclusion

In all the harm that can come from Schwann cell degeneration, there is a far greater potential for what they can create. Schwann cells play a significant role in the pathology of the inner ear. Proliferation of Schwann cells in the vestibulo-cochlear nerve defines the etiology of hearing loss in diseases such as neurofibromatosis type 2 (NF2), while a brief period of demyelination such as in Guillain-Barre Syndrome could also lead to hearing loss. Schwann cells uphold the delicate balance within the inner ear; their range of functionalities carries the converse during dysregulation. Schwann cell and neuron interactions implicated in normal function and disease are rooted in early development where they share a common progenitor. This pliability of Schwann cells makes them a valuable therapeutic target for inducing trans-differentiation or cellular reprogramming to restore neurons within the spiral ganglia.

## Nomenclature

ABR, Auditory Brainstem Response; ASCL1, Achaete-scute homolog 1; bHLH, Basic helix-loop-helix; CMT1A, Charcot-Marie-Tooth Disease Type 1A; CNS, Central Nervous System; c-Jun, Cellular Jun proto-oncogene; DPOAE, Distortion Product Otoacoustic Emissions; ERBB2/HER2, v-erb-b2 avian erythroblastic leukemia viral oncogene homolog 2; GDNF, Glial-Derived Neurotrophic Factor; GBS, Guillain-Barre Syndrome; GPCR, G Protein-Coupled Receptor; GSK, Glycogen Synthase Kinase; HDAC, Histone Deacetylase; HHL, Hidden Hearing Loss; HMG, High Mobility Group; HMG2A, High Mobility Group A2; KROX20/EGR2, Early Growth Response 2; Let7, Lethal-7-microRNA; Lin28, Lin-28 Homolog A; LZTR1, Leucine Zipper Like Post-Translational Regulator 1; MAG, Myelin-Associated Glycoprotein; MBP, Myelin Basic Protein; mTORC1, Mammalian Target of Rapamycin Complex 1; NEUROD1, Neuronal Differentiation 1; NGFR, Nerve Growth Factor Receptor; NGN1, Neurogenin1; NF-κB, Nuclear Factor kappa B; NF2-SWN, Neurofibromatosis Type 2 associated Schwannomatosis; NRG1, Neuregulin 1; NT3, Neurotrophic Factor 3; PAX6, Paired Box Gene 6; P0, myelin protein zero; Plp1, Proteolipid Protein 1; PNS, Peripheral Nervous System; POU3F1/OCT6, POU Class 3 Homeobox 1; POU3F2/BRN2, POU Class 3 Homeobox 2; SGN, Spiral Ganglion Neuron; SMARCB1, SWI/SNF-related BAF chromatin remodeling complex subunit B1; SNHL, Sensorineural Hearing Loss; SOX10, SRY-related HMG Box 10; SOX2, SRY-related HMG Box 2; SRY, Sex-determining Region Y; TNF, Tumor Necrosis Factor; VN, Vestibular Neuron; VS, Vestibular Schwannoma; WNT, Wingless-Related Integration Site (Wnt Signaling Pathway).
